# Evaluation of non-vendor magnetic resonance imaging sequences for use in bladder cancer magnetic resonance image guided radiotherapy

**DOI:** 10.1016/j.phro.2023.100481

**Published:** 2023-08-16

**Authors:** Joan Chick, Sophie Alexander, Trina Herbert, Robert Huddart, Manasi Ingle, Adam Mitchell, Simeon Nill, Uwe Oelfke, Alex Dunlop, Shaista Hafeez

**Affiliations:** a The Joint Department of Physics at The Institute of Cancer Research & The Royal Marsden NHS Foundation Trust, Downs Road, Sutton SM2 5PT, UK; b The Institute of Cancer Research & The Royal Marsden NHS Foundation Trust, Downs Road, Sutton SM2 5PT, UK; c The Royal Marsden NHS Foundation Trust, Downs Road, Sutton SM2 5PT, UK

**Keywords:** MRI sequence evaluation, Non-vendor sequences, MRI guided radiotherapy, Adaptive radiotherapy, Bladder cancer, Quality assurance

## Abstract

Hybrid systems that combine Magnetic Resonance Imaging (MRI) and linear accelerators are available clinically to guide and adapt radiotherapy. Vendor-approved MRI sequences are provided, however alternative sequences may offer advantages. The aim of this study was to develop a systematic approach for non-vendor sequence evaluation, to determine safety, accuracy and overall clinical application of two potential sequences for bladder cancer MRI guided radiotherapy. Non-vendor sequences underwent and passed clinical image qualitative review, phantom quality assurance, and radiotherapy planning assessments. Volunteer workflow tests showed the potential for one sequence to reduce workflow time by 27% compared to the standard vendor sequence.

## 1 Introduction

Magnetic Resonance Image guided radiotherapy (MRIgRT) uses the enhanced soft-tissue image quality of MRI to more confidently guide and adapt Radiotherapy (RT). The availability of hybrid systems that combine a Magnetic Resonance (MR) scanner with a linear accelerator (MR-linac) is increasing. Such systems allow imaging and replanning according to target and OAR anatomy at each fraction [1], [2].

MRI sequences are provided for MR-linac workflows by the vendors; however, these are often designed for use over multiple anatomical areas and may not be fully optimised for the specific target site in question [3], [4]. Alternative sequences may offer advantages such as improved image contrast, faster acquisition or motion assessment information, and new sequences have already been proposed for use in MR-linac workflows [5], [6], [7], [8]. International derived consensus guidance is available for RT-specific MRI protocol optimisation, and gives recommendations for receiver bandwidth, resolution, and acquisition types [9], [10]. However specific guidance for MRIgRT systems is not yet available, and furthermore, using a non-vendor provided sequence may require working outside of the manufacturer’s instructions for use and hence be considered ‘off-label’ use. This requires documented risk-assessment and informed patient consent [11].

MRIgRT is recognised to have great potential to improve bladder cancer treatments, by correcting for inter-fraction variation in bladder position and size due to variable filling [12]. However, a challenge of this approach is increased intra-fraction bladder filling due to longer MRIgRT workflow time compared to C-arm Linac delivery [13]. Hence it is desirable to minimise workflow time as far as practical, making this an appealing site for non-vendor sequence testing.

The aim of this project was to develop a systematic procedure to support risk-assessed non-vendor sequence use in bladder MRIgRT.

## 2 Materials and methods

**Fig. 1:**
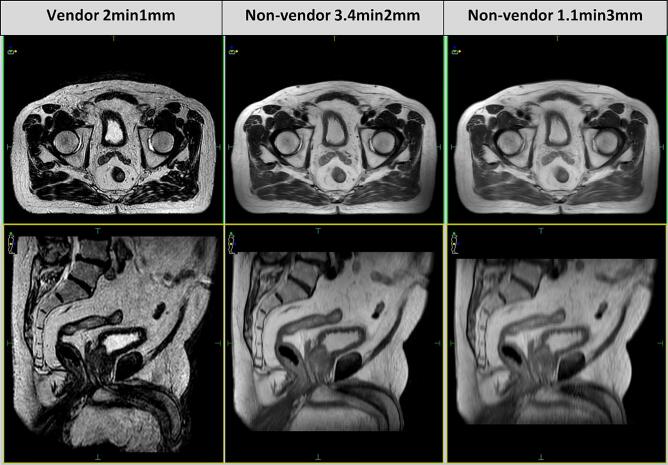
Comparison of non-vendor sequences against the vendor-approved ‘Pelvis’ sequence for MRIgRT workflows. Top: axial, Bottom: sagittal.

A multidisciplinary team (MDT) was assigned to this project, consisting of MR-linac Therapeutic Radiographers, Medical Physicists, and Clinical Oncologists. Two non-vendor sequences were identified for testing, due to their potential to maintain or improve image quality whilst reducing data transfer or image acquisition times. They were named 3.4min2mm and 1.1min3mm, denoting acquisition time and slice thickness, the comparator vendor-approved sequence was 2min1mm, shown in Fig. 1. All data was from patients consented to additional research imaging within the PERMIT (NCT03727698) study on the Elekta Unity MR-linac (Elekta AB, Stockholm, Sweden).

### 2.1 Parameter assessment

Sequences were saved on the MR console with a reference weight of 70Kg to allow standardised Specific Absorption Rate values to be reported. Scanning parameters were set to ensure that non-vendor sequences run within the ‘Normal’ mode of operation, with ‘First level’ only accessible after additional risk assessment [11]. All key sequence parameters were recorded and assessed by an MR experienced Medical Physicist. A checklist ensured that the sequence had the following mandatory parameters: 3D sequence, distortion correction and shim. It also documented any known rationale for sequence modifications as compared to the vendor-approved sequence. The receive bandwidth was documented as Hz/mm and as the fat water shift in mm to compare against RT guidelines and with the vendor-approved sequence [9].

### 2.2 Phantom quality assurance

Images were acquired of the large American College of Radiology (ACR) phantom and used to assess geometric accuracy, image uniformity and ghosting, based on the ACR Quality Assurance (QA) guidance for methods and result criteria [14]. Geometric accuracy measures the diameter of the phantom in four directions and compares against the known value. Image uniformity measures the uniformity of image intensity over a large homogenous region of the phantom. Ghosting assesses the level of background signal arising from ghosting artefacts compared to the true image.

### 2.3 Treatment planning system assessment

Tests were performed within the Treatment Planning System (TPS) to ensure that treatment planning on the new sequence was suitable, most notably for the increased slice thickness compared to the default 1 mm as in the vendor-approved sequence. The three main aspects of the TPS assessment were: deformable image registration, margin expansions to create Planning Target Volumes (PTVs) and plan optimisation based on the site-specific RT protocol.

The assessment was carried out on previously treated clinical data for which the vendor-approved sequence was used for adaptive planning, with existing approved contours and RT plans. The original image data was reformatted with the new slice thicknesses and imported into the TPS. For the Elekta Unity, this was done using VolumeView (Philips, Best, Netherlands) on the MR console and then imported into Monaco (5.40, Elekta AB, Stockholm, Sweden).

Firstly, a deformable image registration was carried out in the TPS between the simulated and original image data, and the propagated external and bones assessed for gross errors. Secondly, contours were copied rigidly to the simulated data, and all planning structures grown following clinical margin recipes. Lastly, treatment plans were optimised and calculated on the simulated data, and clinical goals and dose distributions visually compared with the clinical plans. To aid comparison, the treatment plans were also recalculated on the original data and dose subtractions generated. This process was repeated for three data sets to reflect the variability of clinical cases seen.

### 2.4 Qualitative review

Following initial sequence safety review, images with the new sequences were acquired from three patients, with the standard vendor-approved sequence for the site acquired as part of the treatment workflow. Four reviewers (Clinical Oncologists and MR-linac Therapeutic Radiographers) performed independent assessment.

An image review template was developed in Microsoft forms to guide qualitative review. Four workflow tasks were identified: image registration, clinical target volume (CTV), gross tumour volume (GTV) (if applicable) and organ at risk (OAR) delineation. A four-point Likert scale was used to rate the ease of completing the tasks using the new sequence compared to the vendor-approved sequence: more difficult, same, a little easier and much easier. This Likert scale was established and agreed by the MDT.

Visualisation of OAR and target anatomy was scored independently on a second four-point Likert scale to grade structure visibility as: not visible, unclear, clear or very clear. This Likert scale aligned with trial scoring systems (PRIMER NCT02973828) familiar to assessors. To aid comparison, the vendor-approved sequence was made available to view alongside and/or fuse with the new sequences, but it was not formally scored.

To complete each qualitative review assessors were asked “If the standard vendor-approved sequence is rated as 5 stars (out of 10), how would you rate this sequence in comparison?”

### 2.5 Workflow tests

To quantify the effect of the 1.1min3mm sequence on overall treatment session time and gain acquisition experience, online workflows were completed, initially with a phantom and then a patient volunteer. Timing of workflow steps were recorded, including session image acquisition, automatic registration, plan optimisation, plan check and verification imaging. To facilitate a fair comparison, no contour modification was carried out. Total workflow time was measured from start of session image acquisition to beam on.

## 3 Results

Results for the two sequences reviewed for bladder MRIgRT are shown in Table 1. Both sequences passed phantom QA and parameter review, with higher bandwidth than the vendor sequence (and notably with fat water shift < 1 mm). All tests within the TPS passed, with the observation that when the protocol margin expansions were non-integer multiples of the slice thickness, the effective TPS margin differed superiorly and inferiorly from the protocol margin (marked with * in Table 1). However, all clinical goals were met when the plans were recalculated on the vendor-sequence, with acceptable visual dose distributions.

**Table 1:** Summary and outcomes of non-vendor sequence assessment for bladder MRIgRT. Parameter Assessment: Note not all parameters are detailed here, just pertinent contrast and resolution values. TPS Assessment: Note that where vendor sequence result is n/a, this reflects that these results are not required due to the comparative nature of tests. Margin results marked with * highlight those that differ from protocol margin.

		**Vendor 2min1mm**	**Non-vendor 3.4min2mm**	**Non-vendor 1.1min3mm**
**Parameter Assessment**	Sequence Type	TSE	TSE	TSE
	TSE factor	114	90	90
	Echo Time (ms)	278	87	70
	Repetition Time (ms)	1400	1300	1300
	Flip Angle (°)	90	90	90
	Number of signal averages	1	2	2
	Parallel Imaging	SENSE, 3.6 RL	SENSE, 3.7 RL	SENSE, 4 RL
	Rec voxel size (RL × AP × SI, mm)	0.8 × 0.8 × 1	1 × 1 × 2	0.8 × 0.8 × 3
	Number of slices	300	125	77
	3D sequence	PASS	PASS	PASS
	3D distortion correction	PASS	PASS	PASS
	Automatic shim	PASS	PASS	PASS
	Bandwidth	496 Hz/mm	631 Hz/mm	628 Hz/mm
	Fat water shift (1.5 T)	0.45 mm	0.36 mm	0.36 mm
**Phantom QA**	Geometric accuracy (<1%)	0.08 %	−0.15 %	−0.04 %
	Image uniformity (>87.5%)	93.2 %	87.8 %	87.9 %
	Ghosting ratio (<0.025)	0.000	0.002	0.005
**Treatment Planning System Assessment**	Deformable image registration	PASS	PASS	PASS
	Margin growth (15 mm sup)	15 mm	14 mm *	15 mm
	Margin growth (5 mm inf)	5 mm	6 mm *	6 mm *
	Plan optimisation	n/a	PASS	PASS
	Clinical goals met	n/a	PASS	PASS
	Clinical goals met (recalculated on vendor sequence)	n/a	PASS	PASS
	Visual dose distribution assessment	n/a	PASS	PASS
**Qualitative review**	Workflow suitability (% rated ‘same’ or ‘little easier’)	n/a	93 %	75 %
	Image Quality (% rated ‘clear’ or ‘very clear’)	n/a	95 %	84 %
	Overall score	5/10	5/10	4/10
**Workflow**	Phantom total workflow time	10 min 59sec	n/a	7 min 17sec
	Volunteer total workflow time	20 min 59sec	n/a	15 min 15sec

The 3.4min2mm sequence scored 93% and 95% for workflow suitability and image quality, higher than the 1.1min3mm sequence score of 75% and 84% respectively. The overall qualitative score for 3.4min2mm was 5/10, equal to that of the vendor sequence, with the 1.1min3mm sequence rated as 4/10.

Phantom and volunteer total workflow time for the 1.1min3mm sequence was reduced by 3min42sec and 5min44sec (34% and 27% respectively), compared to using the vendor sequence.

## 4 Discussion

A systematic approach for assessing non-vendor sequences for use on MRIgRT platforms has been introduced and illustrated with an example from bladder cancer RT. Both sequences evaluated were shown to be accurate, safe, and facilitated clinically acceptable treatment plans in the TPS, with the 1.1min3mm sequence offering reduced workflow time whilst maintaining sufficient image quality. Results were documented to provide evidence to support the safe use of non-vendor supplied sequences in clinical workflows.

Workflow assessment was not conducted for 3.4min2mm, as the qualitative review did not offer any justification for the increased acquisition time over the vendor sequence. Despite the lower overall qualitative review score for 1.1min3mm, the shorter acquisition time advocated workflow assessment, to quantify the overall effect on treatment time. Workflow time for the 1.1min3mm sequence was reduced substantially above the time attributed to reduced image acquisition alone. Further time savings were due to smaller imaging data size and quicker data transfer steps within the workflow, also noted in previous studies [8]. As the 1.1min3mm sequence offered workflow time reduction whilst maintaining sufficient image quality, it was approved for clinical use by senior delegates from all professions within the MDT. The 1.1min3mm sequence is now the routine sequence for bladder MRIgRT at our institution.

Initial data from the first 3 patients (51 fractions) treated using the non-vendor sequence showed a reduction in workflow time, from initial imaging to beam on of 28% (7 min 14sec), compared to the previous 5 patients (93 fractions) treated with the vendor sequence. Total in-room time, including patient set up and treatment delivery, also reduced, with the overall percentage of fractions delivered within 30 min rising from 17 to 82%. This is closer to non-MRIgRT bladder treatment times [15], enabling increase of MR-linac capacity and an enhanced patient experience, as patient discomfort grows with increasing time on the treatment couch. There is also the potential to reduce margins to account for reduced intra-fraction filling [15].

Methods describe in this work for bladder MRIgRT, can easily be modified for other treatment sites, and alternative sequences including those used for MRI simulators for non-adaptive RT planning. Each step within the methods should be considered and adapted for the sequence and site in question. Not all methods will need to be repeated for every combination, and as experience with non-vendor sequences matures, the time taken for such assessments should reduce.

Parameter assessment should be carried out for all sequences ensuring that the receive bandwidth complies with recommendations [9], [10]. Phantom QA is essential to confirm image quality and artefact level. Moreover, as the field and application of MRIgRT develops, phantom QA will need to expand to include additional aspects, in particular motion. For example, 4D motion phantoms have been used to verify image reconstruction for motion-robust radial MRI, and navigated sequences [5], [16] and digital phantoms have been used to verify motion information and reconstruct mid-position images [7].

TPS assessment was included to evaluate the complete workflow for an end-to-end evaluation of the non-vendor sequence, it usefully highlighted the margin behaviour for the increased slice thickness. This was communicated to all clinicians involved and could help inform margin decisions for future RT protocols. The decision to base the TPS assessment on simulated data removed confounding image contrast, motion artefacts and anatomy changes. This approach is justified for margin and plan assessment due to the synthetic CT approach for MR adapted plans; image contrast will only affect plan optimisation indirectly via improved contouring which is considered in the qualitative review. Using simulated data to assess the deformable registration is a limitation, however deformation of clinical images was tested as part of the patient workflow, and further investigation could be carried out for other non-vendor sequences if required. It is noted that if the non-vendor sequence has identical image resolution to the vendor sequence, then TPS assessment could be simplified to remove the margin assessment and plan optimisation. For the bladder RT protocol, existing clinical data existed which was used as gold standard for the plan comparison however if this is absent, it is recommended that plans are created on the vendor-approved sequence with the new RT protocol used in this assessment.

The qualitative review assessment was designed to reflect the purpose of the images within the bladder MRIgRT workflow. Other studies use similar qualitative reviews tailored to their workflow under investigation, including Likert or scoring scales aligned with our methodology [5], [8], [17], [18]. Our approach was chosen to facilitate efficient assessment and reduce review ambiguity. Imaging artefacts were not assessed, as initial experience with the non-vendor sequences raised no concern, however they should be considered during qualitative review design [5], [18]. Further quantitative comparisons, such as volume metrics of target contours could also be included [18].

Non-vendor sequences are increasingly being used in MRIgRT, to help realise workflow improvements that are not possible with the current vendor sequences. The process detailed here outlines a systematic end-to-end assessment, illustrating the need for a multi-disciplinary approach. We hope it will give more institutions the confidence to safely expand their imaging capability, despite working ‘off-label’ from the vendor’s advice.

## CRediT authorship contribution statement

**Joan Chick:** Conceptualization, Investigation, Formal analysis, Methodology, Visualization, Writing – original draft, Writing – review & editing. **Sophie Alexander:** Conceptualization, Investigation, Formal analysis, Methodology, Visualization, Writing – original draft, Writing – review & editing. **Trina Herbert:** Conceptualization, Investigation, Formal analysis, Methodology, Writing – review & editing. **Robert Huddart:** Investigation, Writing – review & editing. **Manasi Ingle:** Investigation, Writing – review & editing. **Adam Mitchell:** Conceptualization, Methodology, Writing – review & editing. **Simeon Nill:** Conceptualization, Writing – review & editing. **Uwe Oelfke:** Supervision, Writing – review & editing. **Alex Dunlop:** Supervision, Conceptualization, Investigation, Formal analysis, Methodology, Writing – review & editing. **Shaista Hafeez:** Supervision, Conceptualization, Investigation, Writing – review & editing.

## Declaration of Competing Interest

The authors declare the following financial interests/personal relationships which may be considered as potential competing interests: The Royal Marsden Hospital and The Institute of Cancer Research are members of the Elekta MR-linac Consortium, which aims to coordinate international collaborative research relating to the Elekta Unity (MR-linac). Elekta (Elekta AB, Stockholm, Sweden) and Philips (Philips, Best, the Netherlands) are commercial members of the MR-linac Consortium. Elekta financially supports consortium member institutions with research funding, education and travel costs for consortium meetings. S. Hafeez is bladder tumour site group lead within the MR-linac Consortium. No commercial financial support was received from any organisation for the submitted work.
